# Plant community and soil conditions individually affect soil microbial community assembly in experimental mesocosms

**DOI:** 10.1002/ece3.3734

**Published:** 2017-12-20

**Authors:** Aspen T. Reese, Kyrstin Lulow, Lawrence A. David, Justin P. Wright

**Affiliations:** ^1^ Department of Biology Duke University Durham NC USA; ^2^ Department of Molecular Genetics and Microbiology Duke University Durham NC USA

**Keywords:** aboveground–belowground feedbacks, microbial diversity, old field

## Abstract

Soils harbor large, diverse microbial communities critical for local and global ecosystem functioning that are controlled by multiple and poorly understood processes. In particular, while there is observational evidence of relationships between both biotic and abiotic conditions and microbial composition and diversity, there have been few experimental tests to determine the relative importance of these two sets of factors at local scales. Here, we report the results of a fully factorial experiment manipulating soil conditions and plant cover on old‐field mesocosms across a latitudinal gradient. The largest contributor to beta diversity was site‐to‐site variation, but, having corrected for that, we observed significant effects of both plant and soil treatments on microbial composition. Separate phyla were associated with each treatment type, and no interactions between soil and plant treatment were observed. Individual soil characteristics and biotic parameters were also associated with overall beta‐diversity patterns and phyla abundance. In contrast, soil microbial diversity was only associated with site and not experimental treatment. Overall, plant community treatment explained more variation than soil treatment, a result not previously appreciated because it is difficult to dissociate plant community composition and soil conditions in observational studies across gradients. This work highlights the need for more nuanced, multifactorial experiments in microbial ecology and in particular indicates a greater focus on relationships between plant composition and microbial composition during community assembly.

## 1 INTRODUCTION

Soil microbial communities are among the most diverse microbiomes on the planet (Curtis, Sloan, & Scannell, 2002; Wardle, 2002), reflecting great heterogeneity within and between sites. These communities are critical for ecosystem functioning including nutrient acquisition, nutrient cycling, and soil formation (van der Heijden, Bardgett, & van Straalen, 2008). Through their ecosystem effects, as well as direct interactions, soil microbes play an important role in determining plant productivity and diversity (Klironomos et al., 2011; van der Heijden et al., 2008). Because of this functional importance, as well as their diversity, we need an improved understanding what processes shape soil microbial communities and, in particular, how they assemble.

To date, efforts have been applied to describe relationships between alpha‐ and beta‐diversity of soil microbial communities with environmental parameters. Alpha diversity of soil bacteria has been associated with edaphic conditions, most notably pH (Fierer & Jackson, 2006), but not plant diversity or composition (Fierer & Jackson, 2006; Prober et al., 2014; Wardle, 2006). The evidence for other predicted diversity relationships is more mixed, for example, elevational gradients have been observed (Bryant et al., 2008) and not observed (Fierer et al., 2011).

There are often multiple factors that structure community beta diversity in soils. Bacterial composition is associated with edaphic conditions including pH (Fierer & Jackson, 2006; Hogberg, Hogberg, & Myrold, 2007; Lauber, Hamady, Knight, & Fierer, 2009; Lauber, Strickland, Bradford, & Fierer, 2008; Rousk et al., 2010), C mineralization rates (Fierer, Bradford, & Jackson, 2007), and C:N (Hogberg et al., 2007). But pH seems to explain the most variation in surveys across continental scales (Fierer & Jackson, 2006; Rousk et al., 2010). Little is known about archaea in soils and how they may or may not behave like bacteria (although see Leff et al. (2015) for discussion of archaeal responses to N fertilization).

Biotic factors, in particular the plant community composition, can also play a role in shaping microbial community composition—both directly and indirectly by altering edaphic conditions (van der Heijden et al., 2008; Wardle et al., 2004). Significant relationships between plant and bacterial composition are often found. Within biomes, plant communities often predict microbial beta diversity more than edaphic factors (Barberán et al., 2015; Mitchell et al., 2010; Prober et al., 2014). It is, of course, possible for both abiotic and biotic factors to shape bacterial community composition conjointly (e.g., Barberán et al., 2015; Berg & Smalla, 2009; Marschner, Crowley, & Yang, 2004; Mitchell et al., 2010; Prober et al., 2014), but in some studies, only one or the other dominates (e.g., Cline & Zak, 2015). These inconsistencies may reflect site specificity of variation or differences in the scale at which each force structures communities.

To date, studies on the drivers of microbial composition have primarily relied on surveys of natural communities across pre‐existing gradients to compare the importance of biotic or abiotic drivers (Garbeva, van Veen, & van Elsas, 2004; Girvan, Bullimore, Pretty, Osborn, & Ball, 2003; Nunan et al., 2005). Such observational approaches are limited, though, in their ability to disentangle drivers of patterns from covariation and can also suffer from misdirected causality. Direct tests of the relationships shaping microbial communities are still generally lacking, although recent efforts have begun to corroborate observational research. In particular, experimental fertilization studies have captured consistent responses in microbial biomass, function, and composition, although rarely diversity, in response to nitrogen addition (Frey, Knorr, Parrent, & Simpson, 2004; Leff et al., 2015; Marschner, Kandeler, & Marschner, 2003; Ramirez, Craine, & Fierer, 2012; Ramirez, Lauber, Knight, Bradford, & Fierer, 2010). Explorations of other potential drivers have been limited, primarily focusing on responses to climate change (Castro, Classen, Austin, Norby, & Schadt, 2010; Liang et al., 2015; Waldrop & Firestone, 2006; Zhang et al., 2005) or changes in plant species identity (Innes, Hobbs, & Bardgett, 2004; Marschner, Yang, Lieberei, & Crowley, 2001). Furthermore, experiments manipulating multiple potential drivers are even more rare (but see: Castro et al., 2010; Lee, Kim, Ding, & Kang, 2015; Shen, Xu, Chi, Yu, & Wan, 2014; Marschner et al., 2001). Experimentation has focused primarily on understanding the role of cultivar identity in shaping rhizosphere composition in agricultural soils (e.g., Innes et al., 2004; Marschner et al., 2004; Wieland, Neumann, & Backhaus, 2001), but experiments in bulk soil or nonagricultural systems are less common. The most extensive survey of fertilization effects on grasslands did test for correlations between the magnitude of bacterial response to the soil and plant changes and found that the variation in bacterial composition was directly related to the magnitude of shifts in plant community composition more so than edaphic changes or changes in productivity (Leff et al., 2015). This finding suggests that at a local scale, or under meaningful disturbance regimes, plant community composition may be more important than edaphic characteristics for determining microbial composition.

Here, we used an experimental mesocosm setup to directly assess the relative importance of biotic and abiotic variability in shaping microbial (bacterial and archaeal) community assembly. The mesocosms varied in site across a 13**°** latitudinal gradient, with replicated manipulations of soil conditions and plant composition within sites, to emulate various manifestations of old‐field succession. We employed a fully factorial experimental design with two plant communities and four soil texture treatments. We then collected soil cores to analyze bulk soil microbial composition three and a half years after the mesocosms were installed. This experiment specifically allows us to determine whether edaphic factors or dominant vegetation drive microbial diversity and composition and their relative strength across sites. Furthermore, we tested for specific relationships between different microbial phyla and biotic and abiotic conditions.

## 2 MATERIALS AND METHODS

### 2.1 Experimental design

Parallel experiments were established at five sites across the eastern United States in spring 2012 to explore soil, competition, and climate impacts on old‐field succession. We chose sites that spanned nearly the extent of the Eastern Deciduous Forest (Braun, 1950) and that vary in average temperature (Figure S1). At each site, we set up raised beds (1.52 m inner diameter with 28 cm soil depth) with randomly assigned categorical soil and herbaceous community treatments in a fully factorial design. There were six replicates of each treatment arranged in blocks. The beds had holes drilled in the bottom to allow for drainage but were placed on top of woven landscape cloth to isolate them from the native soil and limit weed growth between beds.

#### 2.1.1 Soil treatments

Prior to addition of vegetation, each bed was filled with a randomly assigned soil treatment. These treatments consisted of a gradient of four soil textures (100% topsoil; 75:25 topsoil: sand; 50:50 topsoil: sand; and 25:75 topsoil: sand), representing the range in soil texture and fertility observed across the latitudinal gradient (Fridley and Wright, 2012). Topsoil was sourced from local commercial sources, due to transportation limitations, and so there were site differences in macro‐ and micronutrient availability. Nevertheless, trends in pH (measured with Hach [Loveland, CO] pH meter), soil moisture (calculated as function of difference between fresh soil mass and oven dried soil mass as a function of fresh soil mass), soil organic matter (calculated as function of difference between oven‐dried soil mass and muffled‐dried soil mass as a function of oven‐dried soil mass), phosphorous (measured in Melich III extracting solution on Beckman‐Coulter [Brea, CA] Spectrophotometer), and cation availability (measured in Melich III extracting solution on atomic absorption spectrometer [Perkin Elmer, Waltham, MA]) measured at the time of experimental setup were generally consistent between the treatments within a site when compared between at all sites (Table S1).

#### 2.1.2 Vegetation treatments

There were three herbaceous community treatments: bunchgrass‐dominated (including *Andropogon virginicus* and *Schizachyrium scoparium*), *Solidago*‐dominated (including *Schizachyrium altissima* and *Schizachyrium nemoralis*), or control plots, which were weeded bi‐annually for the first two years of the experiment and then allowed to seed from the surrounding old field. The vegetation treatments were chosen to emulate two stages in old‐field succession and included native plants present along the latitudinal gradient (Fridley and Wright, 2012). Bunchgrasses were started from locally collected seed in a greenhouse and then shipped as plugs to all sites. *Solidago* rhizomes were collected from native populations and kept on ice and in the dark for shipment to all of the sites for transplant. Pools across all sites with the same herbaceous treatment received the same mix of propagules of species sourced from both northern and southern sites. Herbaceous treatments were randomly assigned and transplant occurred in spring 2012 with a subsequent addition of *Solidago* rhizomes in fall of 2013. Bunchgrass plugs and rhizomes were planted at a density of 25 plants m^2^. In fall 2013, all pools received an addition of woody species seeds.

### 2.2 Assessment of edaphic and vegetation conditions

Plant root simulator (PRS) probes (Western Ag Innovations) were installed in June 2014 and collected 1 month later to measure cation availability in soils following establishment of the plant community treatments. Probes were cleaned with DI water and then shipped to the manufacturer for processing. Concentrations of iron (Fe), magnesium (Mn), cobalt (Cu), zinc (Zn), boron (B), sulfur (S), lead (Pb), aluminum (Al), cadmium (Cd), ammonium (NH_4_^+^), and nitrate (NO_3_^−^) were collected by pooling four anion and four cation probers per plot.

In 2015, (3.5 years after establishment of the pools), surveys were conducted on plant establishment and soil conditions. Edaphic measurements included soil moisture measured with a Hydrosense Probe (Campbell Scientific) and soil carbon to nitrogen (C:N) ratio measured following standard protocols using an elemental analyzer (Carlo Erba). Vegetation measurements included maximum canopy height, photosynthetically active radiation (PAR) at ground level (PAR details) measured with a wand‐type ceptometer (AccuPAR LP‐80, Decagon Devices), aboveground biomass, and belowground root biomass. Aboveground biomass was collected from a 50 × 50 cm quadrat in the center of the each plot, and grasses and forbs were separated and dried at 70°C for 48 hr before weighing. Roots were collected for eight pooled soil cores of 5 cm diameter and 10 cm depth from the same quadrat as the aboveground sample, picked out, and washed then dried at 70°C for 48 hr.

### 2.3 Assessment of microbial community composition and diversity

#### 2.3.1 Sample collection

At the final soil measurement time point (three and a half years after establishment), four pooled soil cores (2.5 cm × 10 cm) were collected for microbial analysis from all grass and *Solidago* pools at all sites between September and December 2015. In addition, we collected soil cores from the control pools at the NC and SYR sites. Cores were stored on ice immediately after collection and preserved at −20°C. The soil corer was cleaned with ethanol between pools. All samples were processed at Duke University.

Microbial community diversity and composition were assessed using targeted amplicon sequencing of the 16S rRNA gene for bacteria and archaea domains. Soil cores were homogenized and passed through a 2‐mm sieve. Total DNA was extracted from ~0.25 g soil using the PowerSoil DNA extraction kit (Mo Bio Laboratories, Inc.) following manufacturer's instructions. The V4 region of the 16s rRNA gene was amplified in triplicate using custom barcoded primers (515f/806r primer pair; Caporaso et al., 2011) then pooled in equimolar ratios, cleaned, and quantified following published protocols (Caporaso et al., 2011, 2012; Maurice, Haiser, & Turnbaugh, 2013). All samples were sequenced in a single MiSeq lane with paired‐end 250 bp reads and reagent kit v2 (Illumina, San Diego, CA). Raw sequence data were processed using scripts from QIIME version 1.8 (Caporaso et al., 2010). OTUs were picked at 97% similarity against the Greengenes reference database (DeSantis et al., 2006). We discarded all samples with fewer than 5,000 reads per sample for insufficient sequence coverage. The 16S rRNA nucleotide sequences generated in this study can be downloaded from the European Nucleotide Archive under study accession number PRJEB14323.

### 2.4 Statistical analyses

Microbial diversity including both archaea and bacterial OTUs was calculated as Shannon diversity, the preferred method for microbial sequence data from complex communities (Haegeman et al., 2013), and OTU richness. We performed Kruskal–Wallis tests to determine whether there were significant differences in diversity among sites. We used linear mixed effect models to test for the effects of soil and herb treatment (fixed effects) with site and block as random effects. Here, we report *p* values of ANOVAs comparing the null and treatment models.

We used permutational multivariate ANOVA (PERMANOVA) to test for significant shifts in overall community composition (including both bacterial and archaeal OTUs) between sites and in response to experimental treatments. A Bray–Curtis dissimilarity matrix computed from square‐root transformed abundance was used to represent community composition. First, we tested for differences due solely to site‐to‐site variation. Then, soil and herb treatments were used as predictor variables with site as “strata,” which limits permutations to within sites, and block as a random effect. The interaction term between soil and vegetation was not found to be significant, and so we did not include it in the final model.

To isolate particular environmental aspects that contributed to microbial variation, we first used linear mixed effect models to test for effects of soil and herb treatment (fixed effects) on environmental parameters with site and block as random effects. Here, we report p values of ANOVAs comparing the null and treatment models with a Bonferroni multiple hypothesis correction. For variables that were significantly impacted by soil and/or herb treatment, we performed PERMANOVA with the variables grouped by class (nitrogen measurements, high abundance cations, low abundance cations, soil moisture and C:N, aboveground measurements, and root biomass) entered as predictor variables and site as “strata.”

To test for differences in relative abundance at higher taxonomic levels, we used linear mixed‐effects models with soil and herb treatment as fixed effects and site and block as random effects. Tests were run on phyla representing on average more than 2% relative abundance. We compared between models including soil treatment and/or herb treatment with a null model only including the random effects. No interaction terms were ever found to be significant. Here, we report *p* values of ANOVAs comparing the null and treatment models with a Bonferroni multiple hypothesis correction. For all phyla, we also conducted Bonferroni‐corrected Spearman correlation tests to determine whether variation in abundance was associated environmental variation (nitrogen measurements, cations, soil moisture, C:N, aboveground measurements, and root biomass). We also performed Kruskal–Wallis tests to determine whether there were significant differences in relative abundance among sites.

We computed the physical distances between pools and compared these to the compositional differences within a site to assess the impact of physical proximity on community similarity. We used a Mantel test to calculate the correlation between the physical distance matrix and the Bray–Curtis dissimilarity matrix computed from square‐root transformed OTU abundance.

All statistical tests were carried out in R (version 3.2.3; R Core Team, 2015). Linear mixed effect models were computed using the lme4 package (Bates, Maechler, Bolker, & Walker, 2015). PERMANOVA was implemented with the “Adonis” function and mantel tests with the “mantel” function in the vegan package (Oksanen et al., 2016).

## 3 RESULTS

**Figure 1:**
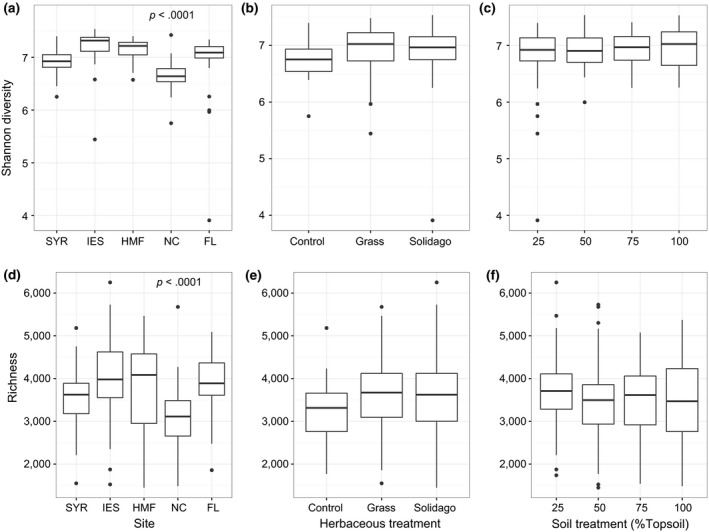
Microbial diversity varies with site but not by treatment. Box plots of microbial Shannon diversity between sites (a), soil treatment (b), and herb treatment (c); richness between sites (d), soil treatment (e), and herb treatment (f). Box plots show quartiles

There was significant between‐site variation in Shannon diversity (*p* < .001, Kruskal–Wallis test; Figure 1a) regardless of treatment. We also observed a greater than fourfold range of richness, which also significantly varied between sites independent of treatment (*p* < .001, Kruskal–Wallis test; Figure 1d). Diversity (Shannon or richness) was not significantly associated with soil (*p* > .05, Figure 1b,e) or herb treatment (*p* > .05, linear mixed‐effects model likelihood tests; Figure 1c,f), however.

**Figure 2:**
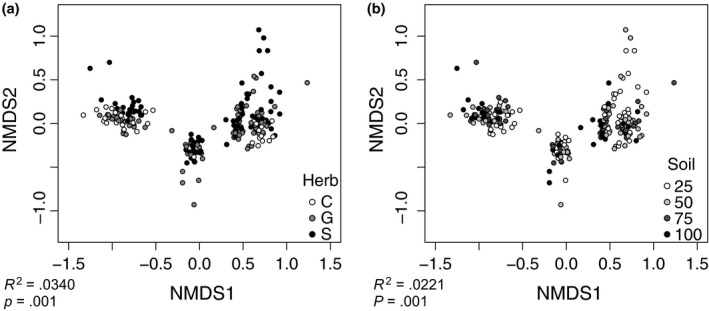
Microbial community composition differs between experimental treatments. Nonmetric multidimensional scaling ordinations illustrating differences between total microbial communities based on herb treatment (a) and soil treatment (b). Points indicate individual beds and are colored by treatment. *R* ^2^ and *p* values refer to PERMANOVA results

In contrast, microbial community composition, calculated as Bray–Curtis dissimilarity between mesocosms, responded significantly to both treatments. While a majority of variation between samples was contributed by site‐to‐site variation (*R* ^2^ = .515, *p* = .001, PERMANOVA; Figure S2), we still observed significant treatment effects once we had controlled for site. Herb treatment explained a greater proportion of the remaining variation (*R* ^2^ = .034, *p* = .001, Figure 2a) than did soil treatment (*R* ^2^ = .022, *p* = .001, PERMANOVA; Figure 2b) when considering variation within sites. We observed no significant interaction between herb and soil treatments.

Multiple abiotic and biotic variables varied significantly due to treatment. Soil moisture and C:N, measured at the end of the experiment, were both significantly associated with soil treatment (*p* < .001, linear mixed‐effects model likelihood tests; Table S2) but not herb treatment. Of the soil variables measured during the experiment, most cations (Mg, Ca, P, Mn, Cu, S, Pb, Al) varied with soil treatment (*p* ≤ .01), K varied with herb treatment (*p* < .001), and two cations (Fe and Zn) responded to both treatments with a significant interaction (*p* < .05, linear mixed‐effects model likelihood tests). Nitrate and total N varied with herb treatment (*p* < .001, linear mixed‐effects model likelihood tests), but ammonia did not respond significantly to either treatment. All biotic variables except photoactive radiation (i.e., aboveground herbaceous and grass biomass, canopy height, and root biomass) were significantly impacted by herb treatment (*p* < .01), with herb biomass also responding to soil treatment with an interaction between the treatment effects (*p* < .05, linear mixed‐effects model likelihood tests).

We next analyzed whether variables that were significantly related to the experimental treatments also associated with microbial beta diversity. We found that K, Mg, Ca, and Cu concentrations were significantly associated with compositional variation (*p* < .05, PERMANOVA; Table S3). In addition, total aboveground biomass and *Solidago* aboveground biomass were also significantly associated (*p* < .05, PERMANOVA). However, there were many significant correlations between environmental parameters, so it is hard to disentangle which are the causative filters acting on microbial composition.

**Figure 3:**
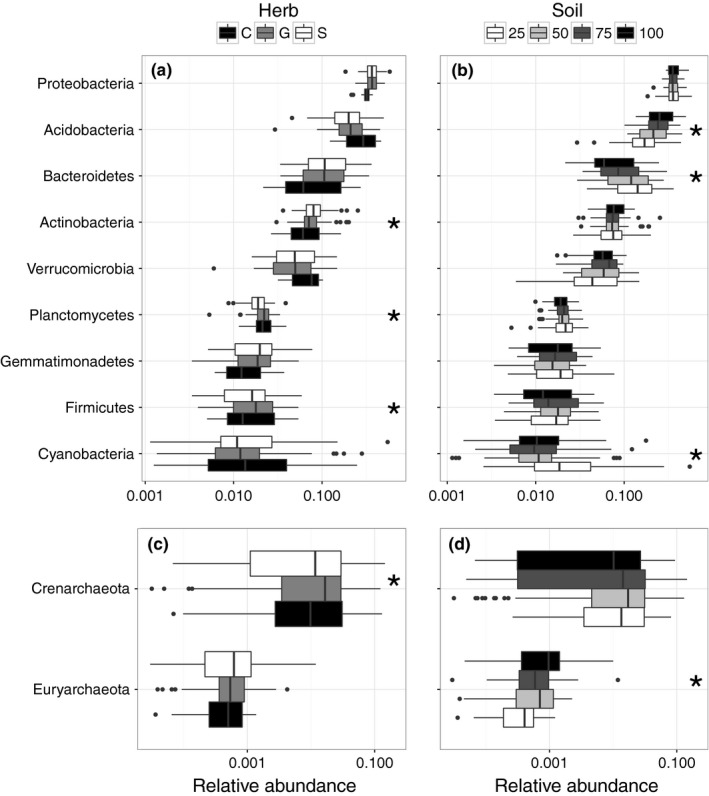
Relative abundance of some higher‐level taxa respond to experimental treatment. Bacterial taxa differ based on herb (a) and soil treatment (b). Archaeal taxa also differ based on herb (c) and soil treatment (d). Box plots show quartiles. Only taxa with greater than 2% average abundance are shown. * indicates *p* < .05

Some of these compositional differences were driven by high‐level taxonomic trends between treatments. Herb treatments were associated with shifts in the relative abundance of Actinobacteria (*p* = .02), Planctomycetes (*p* = .002), and Firmicutes (*p* < .001, linear mixed‐effects model likelihood tests; Figure 3a). Increasing sand content in the beds was associated with higher Bacteroidetes (*p* < .001) and Cyanobacteria (*p* = .03) relative abundance as well as a decrease in Acidobacteria (*p* < .001, linear mixed‐effects model likelihood tests; Figure 3b). No models supported significant interactions between soil and herb treatment. Proteobacteria, the most abundant phylum, Verrucomicrobia, and Gemmatimonadetes were the only other phyla with more than 2% average abundance, but none were associated with soil or herb treatment. All phyla did significantly vary by site (*p* < .001 except Planctomycetes *p* = .02, Kruskal–Wallis tests; Figure S3).

All phyla except Cyanobacteria were correlated with at least one environmental measurement (Table S4). Planctomycetes abundance was only negatively associated with root biomass (*p* = .0001, ρ = −.28, Spearman correlation). The other phyla were associated with between five and thirteen variables (*p* < .001, Spearman correlations). The absolute correlation coefficient values ranged from 0.25 (nitrate was negatively associated with Acidobacteria abundance) to 0.69 (soil moisture was negatively associated with Verrucomicrobia relative abundance). Soil moisture and ammonia were associated with the most bacterial phyla (seven each). Phyla which were significantly impacted by herb or soil treatment were not necessarily correlated with the environmental variables that were significantly impacted by the same treatments. Furthermore, variables that were not associated with either treatment (e.g., ammonia) were still found to be correlated with phyla abundances.

Archaea represented much less of the community (2.15% ± 2.48) than bacteria, but they also varied significantly between sites (*p* < .001, Kruskal–Wallis tests) and treatment. Correcting for site‐to‐site variation (Figure S3), we observed a relationship between total archaeal relative abundance and herb treatment (*p* = .03, linear mixed‐effects model likelihood test). For the two most abundant archaeal phyla, we observed an effect of herb treatment on Crenarchaeota (*p* = .03; Figure 3c) and an effect of soil treatment on Euryarchaeota (*p* = .001, linear mixed‐effects model likelihood test; Figure 3d). Euryarchaeota and Crenarchaeota abundance were both correlated with ammonia and then independently with a number of soil measurements (*p* < .001, linear mixed‐effects model likelihood tests; Table S4). Crenarchaeota abundance was also negatively correlated with root biomass (*p* < .001, rho = −0.5, Spearman correlation).

To determine whether nearby pools were more similar, we compared physical distance matrices to community dissimilarity matrices. At four of the five sites, physical proximity was not associated with increased compositional similarity (*p* > .05, mantel test). At the Syracuse site, physical distance was significantly correlated with square‐root transformed Bray–Curtis dissimilarity (Mantel statistic *r* = .1401, significance = 0.019). This result indicates that beds closer together in space were more similar in community composition.

## 4 DISCUSSION

This work is among the first to report the effects of biotic and abiotic conditions on soil community assembly from a fully factorial experiment. After controlling for the large between site effects, we observed significant influence of both treatments on composition in old‐field soils, with plant community composition explaining slightly more of the variation in beta diversity. In contrast, while we documented site‐to‐site variation, we found no significant influence of soil or herb treatment on alpha diversity.

Microbial community assembly, like all ecological community assembly, is mediated by the forces of selection, dispersal, diversification, and drift (Nemergut et al., 2013; Vellend, 2010). In many ways, microbial assembly resembles plant primary succession (Fierer, Nemergut, Knight, & Craine, 2010), but the impact of dormancy and rapid evolution, as well as the immense diversity of microbes, can elicit unique patterns. Furthermore, the feedbacks between microbes and plants may be critical for the development of both communities (e.g., colonization of plants associated with nitrogen‐fixers in glacial forelands [Matthews, 1992]). In this study, we were able to isolate the effects of plant communities and soil conditions to explore their relative importance for soil bacteria and archaea. Furthermore, the predominant lack of local scale spatial signal (only one of the five sites showed evidence of distance effects on microbial communities) indicates that dispersal between beds was not an important factor, further accentuating the impact of our treatments.

Recently, Cline and Zak (2015) performed the first study to query microbial composition in old‐field soils with amplicon sequencing. They found that that edaphic conditions were more predictive of bacterial community structure than differences in plant communities. However, they employed a space‐for‐time substitution design to assess successional change. The limitations of using such a design (Johnson & Miyanishi, 2008) are particularly acute in studies of microbial composition where site‐to‐site variation can be substantial. Our experiment builds on their insights by independently manipulating biotic and abiotic factors known to matter in old‐field settings and then sampling at a single time point. While we find that edaphic conditions do affect microbial composition in the early stages of old‐field succession, we also found that plant community differences are at least as important. These results are most applicable to old‐field soil communities but the effects may be more general, with additional comparative, experimental work necessary to determine what conditions and under what circumstances matter most.

The mesocosms received microbial inocula that were similar between pools within a site, including microbes sourced from the soil and the transplanted plants. Site‐to‐site variation (the largest variation we observed in this dataset) was likely driven by differences in the local soil conditions and the soil sourced inocula because different, locally sourced, commercial soils were used at each site. In addition, sites varied in climate including temperature and precipitation throughout the experiment, which may have contributed to continued divergence between sites during assembly. Nevertheless, all of our soil communities were dominated by members of eight of the nine phyla that are most often encountered in soil (Janssen, 2006), and so are likely representative of how natural soil microbial communities can respond to abiotic and biotic conditions. Our analyses, which corrected for site‐to‐site variation, were focused on determining the relative impact of those conditions on local assembly of microbial communities.

The variation in microbial composition due to plant community type manifested in both bacterial and archaeal taxa. The three bacterial phyla that responded to vegetation treatment were Actinobacteria, Firmicutes, and Planctomycetes. Actinobacteria are generally considered to be copiotrophic and typically increase in response to soil fertilization (Leff et al., 2015; Ramirez et al., 2010), but here, we observed they were negatively correlated with ammonia levels and positively correlated with soil C:N indicating they may do well in lower nitrogen environments. The plant treatment associated variation here—highest abundance in control pools and lowest abundance in *Solidago* pools—may be related to their life history strategy or other nonresource‐mediated mechanisms. Notably, we did find that they were positively correlated with canopy height and total plant biomass.

The roles of Firmicutes and Planctomycetes in soil are less well understood (Buckley, Huangyutitham, Nelson, Rumberger, & Thies, 2006; Fierer et al., 2007), but our results indicate that future work should focus on their interactions with plant communities. In particular, both phyla were negatively associated with root biomass. Furthermore, experimental *Solidago* invasion in Europe, outside of its native range, has been shown to alter microbial biomass (increasing fungal/bacterial ratio) in wetland soils (Scharfy, Gusewell, Gessner, & Venterink, 2010), but to our knowledge, no research has been conducted on *Solidago* associations with specific soil microbial taxa. Our results suggest that such research would be fruitful. Grass identity has not been found to affect experimental microbial communities (Singh, Munro, Potts, & Millard, 2007), but the difference between grass‐ and forb‐associated microbial community composition has not been tested previously (although see Stephan, Meyer, & Schmid, 2000 for discussion of functional diversity varying between grass and forb communities). The presence of fungi has been shown to alter plant competition between grasses and forbs (Callaway, Mahall, Wicks, Pankey, & Zabinski, 2003; Marler, Zabinski, & Callaway, 1999); our results here suggest that the identity of plants may also mediate interactions between microbes. More generally, because herb treatment explained relatively more of the variation in the composition dissimilarity matrix but was only significant in less abundant phyla, further exploration of the fine‐scale variation in communities in response to changes in plant composition will be necessary.

Crenarchaeota, which were the most abundant archaeal phyla by an order of magnitude, were relatively more common in the grass‐dominated plots. Crenarchaeota include ammonia oxidizers and have been suggested to play important functional roles in carbon metabolism (Kemnitz, Kolb, & Conrad, 2007) and nitrogen cycling in soils (Gubry‐Rangin, Nicol, & Prosser, 2010; Schauss et al., 2009). They are known to respond positively to N addition (Leff et al., 2015), but here, we observed a negative correlation between their relative abundance and ammonia levels. Diversity within the Crenarchaeota has also been shown to increase during primary succession (Nicol, Tscherko, Embley, & Prosser, 2005). That increase was associated primarily with changes in niche availability but not changes in plant species, which differs from our result that abundance of Crenarchaeota is associated with grass (early succession)‐dominated rather than *Solidago* (later succession)‐dominated plots.

The taxonomic responses to soil treatment were observed in an entirely different suite of bacteria and archaea. We might have expected Acidobacteria, a largely oligotrophic phylum, to decrease with increasing N content (i.e., increasing topsoil content; Leff et al., 2015; Ramirez et al., 2010) and Bacteroidetes, a copiotrophic phylum (Fierer et al., 2007), to increase, but we found the opposite. Acidobacteria were negatively correlated with nitrate levels and positively correlated with ammonia, while Bacteroidetes abundance was negatively correlated with ammonia. Euryarchaeota decreased in abundance with increasing sand content and were positively correlated with ammonia levels. This result is contrary to what we might expect given that Euryarchaeota includes N‐fixing methanogens which are at a competitive advantage under low‐nitrogen conditions (Leff et al., 2015). Our results indicate that more complicated patterns than just total nitrogen variation between our soil treatments were likely driving compositional variation in response to soil treatment. Finally, Cyanobacteria were fairly consistent except for a marked increase in abundance in the 75% sand beds, which may be due to increased light availability in the upper soil crust.

Similar to previous work (Wardle, 2006; Prober et al., 2014; e.g., Fierer & Jackson, 2006), microbial diversity did not respond to experimental plant treatments. The lack of soil effect here, which contradicts findings elsewhere (Fierer & Jackson, 2006), may be due to the relatively small experimental effects on pH relative to intersite variation.

None of our analyses identified interactions between the soil and plant treatments on microbial composition. Previous research has found interactions between plant species and soil fertility on microbial functioning (Innes et al., 2004). In contrast, our result indicates that the microbial community composition may be disjunct in its response to drivers, a hypothesis also supported by our finding that none of the phyla exhibiting a significant response was affected by both treatments. Similarly, we often found relationships to individual drivers that were separate from overall treatment effects and associations with environmental factors that are less commonly studied (e.g., cations and light availability). Future experiments may not need to be fully factorial, therefore, but multidriver experiments involving many manipulations will certainly be necessary to identify all the relevant forces structuring microbial assembly. Many experiments to date have focused on N addition (Frey et al., 2004; Leff et al., 2015; Ramirez et al., 2010, 2012) and simulating climate change (increased temperature and CO2, decreased soil moisture; e.g., Waldrop & Firestone, 2006; Castro et al., 2010). These experiments are certainly crucial to predict responses to expected global changes due to human activities. However, our understanding of assembly and structure of these communities is still incomplete enough to require additional basic experiments to supplement the extensive observational biogeography data (Barberán et al., 2015; Fierer & Jackson, 2006; Hogberg et al., 2007). The analyses here were limited by the environmental data available (most notably the absence of individual pool pH data); future experiments could build upon this work by including more physical measurements both at the beginning and during microbial assembly. Furthermore, to go beyond identifying relationships between microbial taxa and environmental variation to actually determining mechanism of control will require more targeted experiments.

Understanding the forces that shape microbial communities from a basic perspective is crucial for understanding global biogeochemical cycles and how they will be impacted by anthropogenic change. Here, we were able to dissociate the effects of changes in biotic and abiotic conditions to identify their separate impacts. Notably, while much research has focused on nutrient addition or other changes in soil conditions, we found that plant community composition explained more variation than edaphic treatment at a local scale. Additional research will be necessary to understand the mechanism by which plants shape microbial composition and functioning in order to resist change under altered climate and nutrient regimes.

## CONFLICT OF INTEREST

None declared.

## AUTHOR CONTRIBUTIONS

ATR and JPW conceived the study; ATR, JPW, and LAD designed methodology; ATR and KL processed samples and analyzed data; ATR and JPW wrote the manuscript**.** All authors discussed the results, commented on the manuscript, and gave final approval for publication.
