# Supplementary material for: Plant community and soil conditions individually affect soil microbial community assembly in experimental mesocosms
Source: Ecol Evol. 2017 Dec 20;8(2):1196–205. doi: 10.1002/ece3.3734 (PMC5773302; doi:10.1002/ece3.3734)
Supplement: Supplementary file 1 [file ECE3-8-1196-s001.pdf]

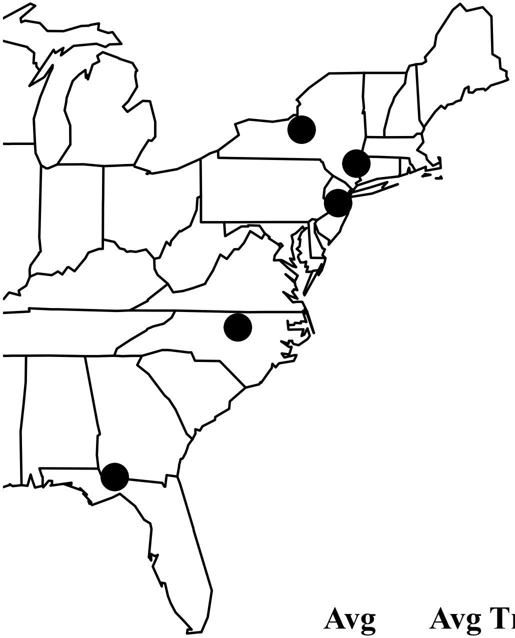

| Site | Location | Latitude | Avg Tmax °C | Avg Tmin °C | Precip (cm) |
|---|---|---|---|---|---|
| SYR | Syracuse, NY | 43° 20' | 13.8 | 3.2 | 104 |
| IES | Millbrook, NY | 41° 47' | 15.8 | 4.4 | 131 |
| HMF | Somerset Co., NJ | 40° 30' | 17.5 | 6.2 | 122 |
| NC | Durham, NC | 36° 00' | 21.6 | 8.6 | 119 |
| FL | Tallahassee, FL | 30° 40' | 26.5 | 13.4 | 139 |
