# Supplementary figures and images for: Plant community and soil conditions individually affect soil microbial community assembly in experimental mesocosms
Source: Ecol Evol. 2017 Dec 20;8(2):1196–205. doi: 10.1002/ece3.3734 (PMC5773302; doi:10.1002/ece3.3734)

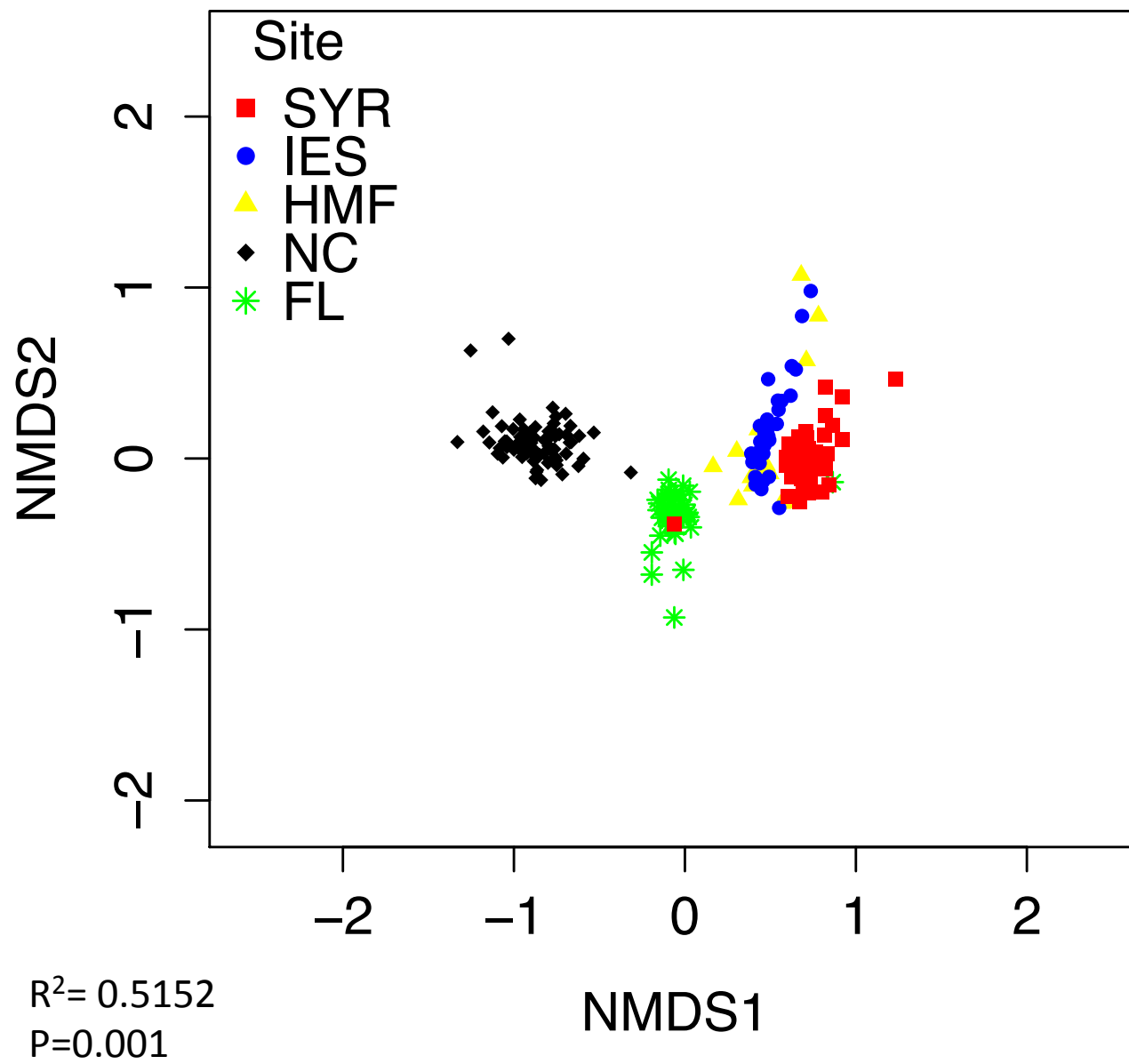

Supplement: Supplementary file 2 [file ECE3-8-1196-s002.pdf]

SYR IES HMF NC FL

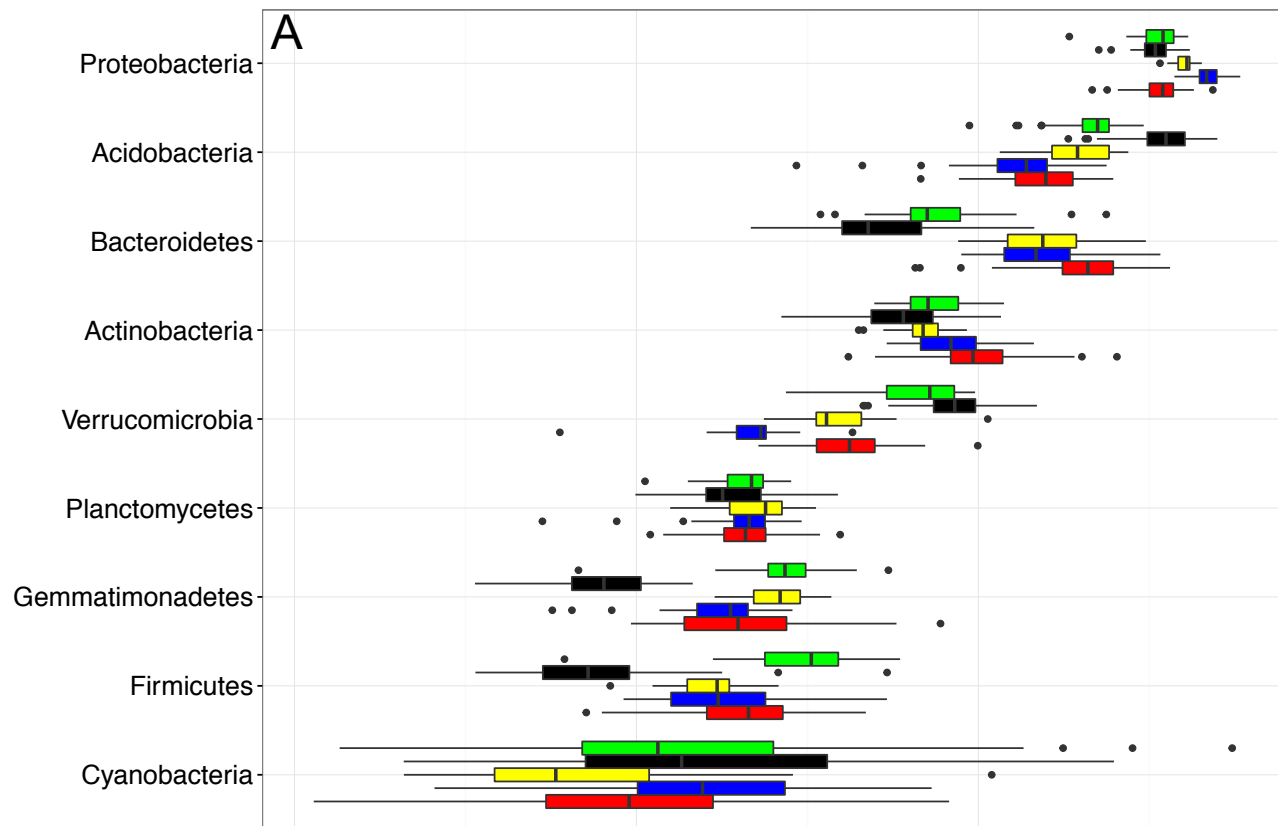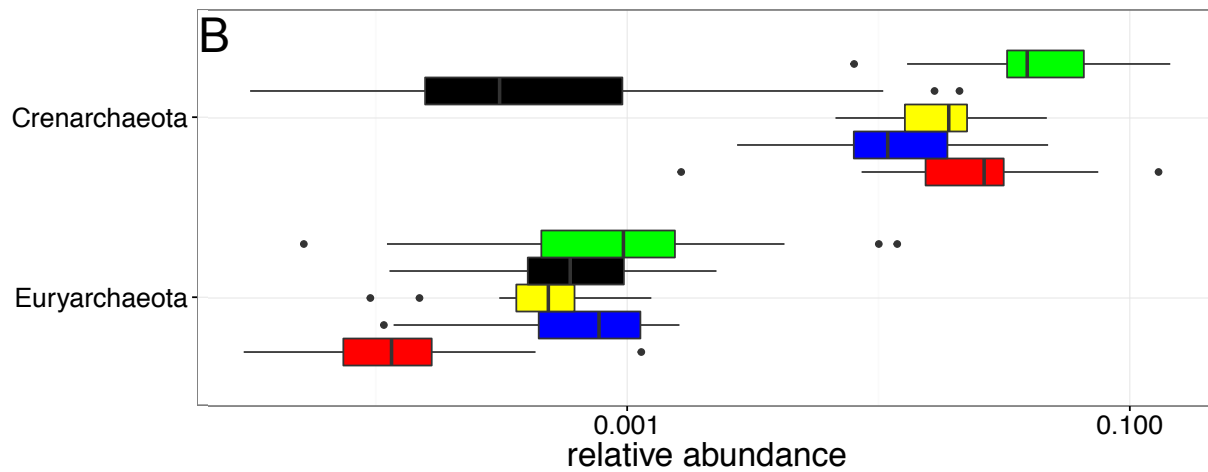

Supplement: Supplementary file 3 [file ECE3-8-1196-s003.pdf]
