# Supplementary material for: Plant community and soil conditions individually affect soil microbial community assembly in experimental mesocosms
Source: Ecol Evol. 2017 Dec 20;8(2):1196–205. doi: 10.1002/ece3.3734 (PMC5773302; doi:10.1002/ece3.3734)
Supplement: Supplementary file 4 [file ECE3-8-1196-s004.xlsx]

Supplementary Table 1: Soil treatments produced consistent trends in variation in edaphic conditions across sites (although site to site variation is much larger than variation due to treatments). All values are for single soil samples from representative pools before vegetation treatments were applied. * indicates value below the detection limit.

Site	% Topsoil	pH	%SM	%SOM	ug P/g soil	ug K/g soil	ug Ca/g soil	ug Mg/g soil
SYR	25	8.2	3.88	1.05	*	99.75	300.74	0.77
	50	8.2	2.41	1.58	2.14	112.66	319.54	0.69
	75	7.9	2.99	2.31	73.15	127.31	32.99	5.66
	100	8.0	6.89	3.47	205.69	141.63	23.71	8.47
IES	25	7.9	5.03	1.10	139.18	116.53	13.79	9.38
	50	7.7	7.69	2.11	187.43	126.39	18.93	8.39
	75	7.6	9.24	2.69	221.35	131.63	17.90	8.84
	100	7.4	11.86	3.98	273.91	157.32	11.25	16.41
HMS	25	7.4	5.27	1.57	332.87	126.72	8.03	16.60
	50	7.3	6.33	2.22	392.52	132.85	9.02	16.15
	75	7.2	9.30	3.04	429.49	145.65	9.59	17.81
	100	6.7	7.67	3.91	492.20	156.46	10.15	18.28
NC	25	5.6	1.14	0.63	23.57	123.71	1.62	45.98
	50	6.0	1.60	1.44	46.03	136.75	3.75	42.95
	75	5.1	1.02	2.00	56.44	147.25	4.14	56.69
	100	5.0	3.40	2.96	67.24	160.74	5.14	60.99
FL	25	6.8	2.37	0.57	251.72	111.79	2.47	13.65
	50	6.5	4.31	1.73	500.60	120.16	5.15	16.31
	75	6.5	4.11	1.99	540.91	118.09	5.84	15.87
	100	6.3	6.00	2.95	617.90	123.60	7.43	16.75
