# Supplementary material for: Plant community and soil conditions individually affect soil microbial community assembly in experimental mesocosms
Source: Ecol Evol. 2017 Dec 20;8(2):1196–205. doi: 10.1002/ece3.3734 (PMC5773302; doi:10.1002/ece3.3734)
Supplement: Supplementary file 8 [file ECE3-8-1196-s008.docx]

Fig S1: Map and climate summary data for field sites (24 year averages [1980-2003] of data from DAYMET US Data Center).

Fig S2: Microbial community composition differs between sites. Nonmetric multidimensional scaling ordination illustrating compositional differences across sites for the total microbial community. Points indicate individual beds and are colored by site. R^2^ and P values refer to PERMANOVA results of site effects.

Fig S3: Relative abundance of all higher-level taxa differ by site. Bacterial taxa (A) and archaeal taxa (B) boxplots show quartiles. Only taxa with greater than 2% average abundance are shown.

Table Legends

Table S1: Soil treatments produced consistent trends in variation in edaphic conditions across sites (although site to site variation is much larger than variation due to treatments). All values are for single soil samples from representative pools before vegetation treatments were applied. * indicates value below the detection limit.

Table S2: Abiotic and biotic variables respond to experimental treatments. Results (Bonferroni-corrected p-values) for ANOVA comparing linear mixed effects models including treatment effects relative to null model only including site effects. Interaction test compares model with both treatment terms to model with more significant single term effect.

Table S3: Community composition is associated with variation in abiotic and biotic variables. Results (p values) for PERMANOVA testing impacts of environmental measurements on Bray-Curtis dissimilarity matrix. Models were run with additive interactions between variables in the clusters indicated.

Table S4: Phyla relative abundance is associated with variation in abiotic and biotic variables. Results (p values, correlation coefficients (rho)) for Bonferroni-corrected Spearman correlation tests analyzing associations between environmental measurements and relative abundance data for phyla representing >2% abundance on average.
